# The Role of hs-CRP and Systemic Immune–Inflammation Index in Sarcopenia and Sarcopenic Obesity in Young Populations: Results from the National Health and Nutrition Examination Survey 2015-2018

**DOI:** 10.2174/0118715303440752251229094023

**Published:** 2026-06-03

**Authors:** Dengfeng Ge, Yue Yu, Jin Rao, Hao Ren, Zhinong Wang, Bin Zhang

**Affiliations:** 1 Department of Cardiothoracic Surgery, Jiangsu Province Hospital of Chinese Medicine,Nanjing University of Chinese Medicine, Nanjing, China;; 2 Department of Cardiothoracic Surgery, Changzheng Hospital, Naval Medical University, Shanghai, China

**Keywords:** Sarcopenia, sarcopenic obesity, high-sensitivity C-reactive protein, systemic immune–inflammation index, NHANES, multivariable logistic regression

## Abstract

**Introduction:**

Inflammation is critical in developing sarcopenia and sarcopenic obesity in older adults, yet evidence in younger populations remains scarce. This study examines associations of high-sensitivity C-reactive protein (hs-CRP) and systemic immune–inflammation index (SII) with sarcopenia and sarcopenic obesity among US adults.

**Methods:**

By using data from the 2015–2018 cycles of the National Health and Nutrition Examination Survey (NHANES), we conducted this cross-sectional analysis of adults aged ≥18 years. Overall, 4,870 participants were included, and both hs-CRP and SII values were divided into quartiles. Multivariable logistic regression was used to analyze the associations of hs-CRP and SII with both sarcopenia and sarcopenic obesity. Interaction tests were conducted to confirm model stability and identify individuals at high risk of developing sarcopenia.

**Results:**

Multivariable logistic regression showed positive associations of SII and hs-CRP with sarcopenia and sarcopenic obesity. Compared to participants in the lowest SII quartile, those in the highest SII quartile had an increased odds of sarcopenic obesity (odds ratio (OR) 1.82, 95% confidence interval (CI) 1.24–2.67, *p* < 0.0022) and sarcopenia (OR 1.94, 95% CI 1.41–2.68, *p* < 0.0001). Participants in the highest hs-CRP quartile were also associated with significantly increased odds of sarcopenic obesity (OR 24.69, 95% CI 8.99–67.78, *p* < 0.0001) and sarcopenia (OR 6.09, 95% CI 3.89–9.53, *p* < 0.0001).

**Discussion:**

Our findings extend the conclusions of previous studies conducted in older populations by demonstrating that systemic inflammation is also positively associated with the incidence of sarcopenia and sarcopenic obesity in younger adults. This finding suggests that individuals with an elevated inflammation level are more likely to develop these conditions. To our knowledge, this is the first study to report these associations in a younger adult population, offering reliable data and practical options for screening sarcopenia in high-risk community groups by using routine clinical tests.

**Conclusion:**

These findings support the potential of hs-CRP and SII as individual or combined screening tools to assess sarcopenia and sarcopenic obesity risk in community or large-population settings.

## 1 INTRODUCTION

Owing to global urbanization, population aging, and lifestyle changes, the prevalence of both sarcopenia and obesity is rapidly increasing, making these conditions significant global public health issues [1-3]. Sarcopenia, defined by muscle loss, refers to a progressive, widespread decline in skeletal muscle mass, strength, and physical function [4-7]. Obesity is a chronic, systemic inflammatory condition caused by excess energy intake, leading to fat accumulation and adverse health effects. The coexistence of sarcopenia and obesity is termed sarcopenic obesity, which was first coined by Baumgartner in 2000 [8, 9]. Globally, approximately 11% of older adults have sarcopenic obesity, and its prevalence increases in those aged >70 years [10].

Currently, there is a lack of global consensus on the diagnostic method for sarcopenia/sarcopenic obesity [11, 12]. Commonly used diagnostic criteria have been proposed by various working groups, including the European Working Group on Sarcopenia in Older People [13-15], International Working Group on Sarcopenia [16], Asian Working Group for Sarcopenia [5], and Foundation for the National Institutes of Health [17]. Most existing criteria suggest defining sarcopenia based on low muscle mass, muscle strength, and/or physical performance. The measurement of muscle mass relies on computed tomography, magnetic resonance imaging, and dual-energy X-ray absorptiometry (DXA) [18]. However, these tools are not practical for large-scale population screening [19]. It is crucial to identify affected individuals and understand the factors that contribute to these conditions [20]. Therefore, there is a need to develop simple and efficient screening tools and assessment criteria for sarcopenia. The development of sarcopenia and sarcopenic obesity in younger populations is a multifactorial phenomenon. Key contributing factors include physical inactivity, inadequate nutrition—particularly low protein intake—which impairs muscle protein synthesis, insulin resistance that disrupts anabolic signaling [21, 22], and hormonal imbalances. Underpinning these mechanisms is the role of chronic, low- grade inflammation.

Previous epidemiological studies have explored the relationship between inflammation and sarcopenia or sarcopenic obesity; the results revealed an association between obesity, sarcopenia, and inflammatory markers. In particular, sarcopenic obesity showed an association with elevated interleukin-6 and C-reactive protein (CRP) concentrations [23, 24]. Previous studies have often relied on data collected decades ago [25, 26] and have mostly focused on older individuals aged >60 years. Although sarcopenia is typically age-related, obesity can occur at any age. However, there is a scarcity of data from younger individuals with sarcopenic obesity, and the relationship between this condition and inflammation in the younger population has largely been neglected [27]. Therefore, there is a need to assess this relationship using more recent data from younger individuals.

This study was a cross-sectional analysis of data from the 2015–2018 cycles of the National Health and Nutrition Examination Survey (NHANES). Through this study, we aim to determine the association between sarcopenia and sarcopenic obesity in adults aged ≥18 years by using two easily measurable inflammatory biomarkers commonly used in clinical trials, namely high-sensitivity CRP (hs-CRP) and the systemic immune–inflammation index (SII).

## 2 MATERIALS AND METHODS

### 2.1 Study Design

This was a cross-sectional study using data from the NHANES, which is designed to assess the health and nutritional status of both adults and children in the United States [28]. Conducted by the Centers for Disease Control and Prevention, the NHANES protocol has been approved by the Research Ethics Review Board of the National Center for Health Statistics. Written informed consent was obtained from all participants. The data generated and/or analyzed in this study are publicly available on the official NHANES website (https://www.cdc.gov/nchs/nhanes/index.html).

### 2.2 Study Population

We used the data of consecutive NHANES cycles (2015–2018). These survey cycles provided comprehensive information on SII and its association with sarcopenia and sarcopenic obesity [29]. A total of 19,225 participants were recruited for this cross-sectional study. After excluding participants aged <18 years (n = 7,377) and with missing data on hs-CRP (n = 1,332), SII (n = 33), body mass index (BMI) (n = 578), DXA (n = 4,913), and other data (n = 122), 4,870 participants were included in the analysis. The sample size was determined based on the availability of complete data in the NHANES database, which is a nationally representative sample designed to ensure statistical power for subgroup analyses. No additional sample size calculation was performed as this is a secondary analysis of publicly available data. The use of these data was approved by the Research Ethics Review Board of the National Center for Health Statistics, and all participants provided written informed consent. The flowchart of participant selection is shown in Fig. (**1**).

### 2.3 Inflammatory Biomarkers

During participant interviews, blood samples were collected to measure hs-CRP concentration and SII, which were used as indicators of inflammation [30]. We selected hs-CRP and SII as inflammatory biomarkers because they are easily measurable by routine clinical blood tests, improving the clinical applicability of the findings. By following standard procedures, blood was drawn from the participants through venipuncture after a 9-h fast at mobile examination centers. Serum samples were analyzed by the latex-enhanced turbidimetric immunoassay method to quantify hs-CRP concentration, which was divided into quartiles as follows: <0.67 mg/L (reference), ≥0.67 to <1.63 mg/L, ≥1.63 to <3.83 mg/L, and ≥3.83 mg/L. SII was calculated using the following formula: (platelet count × neutrophil count) ÷ lymphocyte count [31], and it was also categorized into quartiles as follows: <319.42 × 10^9^ cells/L (reference), ≥319.42 to <441.67 × 10^9^ cells/L, ≥441.67 to <609.08 × 10^9^ cells/L, and ≥609.08 × 10^9^ cells/L [32].

### 2.4 Sarcopenia and Sarcopenic Obesity

DXA was used to measure appendicular skeletal muscle mass (ASM), which represents the total lean muscle mass in the arms and legs [33]. DXA cannot be used in individuals who are pregnant, taller than 192.5 cm, or weighing more than 136.4 kg [34-36]. Therefore, participants who met any of these criteria were excluded from the sarcopenia/sarcopenic obesity analysis. The sarcopenia index was defined as the ASM to BMI ratio. Sarcopenia was defined as a sarcopenia index of <0.512 for women and <0.789 for men [37]. Sarcopenic obesity was defined as the presence of both sarcopenia and obesity. In this study, obesity was defined as a BMI of ≥30 kg/m^2^ for both men and women [38].

### 2.5 Covariates

Covariates were selected based on previously published literature [39-42]. Demographic data included sex, age, race, educational attainment, poverty-to-income ratio (PIR), and marital status. PIR > 4 was defined as high income, PIR ≥ 1 to ≤ 4 was defined as medium income, and PIR < 1 was defined as low income [43-45]. BMI was obtained from the physical examination data. Peripheral blood platelet, neutrophil, and lymphocyte counts were retrieved from the laboratory data. The following four comorbidities were considered for determining participant eligibility: (1) cardiovascular disease (CVD), including history of myocardial infarction, congestive heart failure, stroke, and coronary heart disease (CHD), (2) hypertension, (3) diabetes mellitus, and (4) chronic kidney disease (CKD). The diagnosis of these conditions was based on previously published diagnostic criteria [46, 47], which align with the measurement indicators in the NHANES database.

### 2.6 Statistical Analyses

The sample weights of the individuals were determined according to the NHANES recommended sample weight of Subsample 2 Year MEC Weight (WTMEC2YR) ÷ 2 [48, 49]. To enhance statistical efficiency and facilitate clinical application owing to the high absolute SII values, we divided the SII values by 100 for the purpose of the analysis. With this adjustment, an increase of 1 unit in SII corresponded to an absolute increase of 100 [50]. The characteristics of the participants were summarized by overall demographics and inflammatory biomarker quartiles (hs-CRP and SII quartiles).

Bivariate logistic regression analysis was used to evaluate the association between the inflammatory biomarkers (hs-CRP and SII) and sarcopenia/sarcopenic obesity. To further assess the stability of the relationship between the inflammatory biomarkers and these two conditions, we constructed three regression models, with gradual adjustment for confounding factors. Model 1 was unadjusted; Model 2 was adjusted for age, sex, and race; and Model 3 was adjusted for marital status, educational attainment, PIR, smoking status, diabetes mellitus, CKD, hypertension, CHD, and hyperlipidemia [51, 52]. To evaluate the robustness of the primary analyses, sensitivity analyses were performed by categorizing the inflammatory biomarkers (hs-CRP and SII) into quartiles. We calculated odds ratios (ORs) and 95% confidence intervals (CIs) for the highest *versus* lowest quartiles of hs-CRP and SII in relation to sarcopenia and sarcopenic obesity by using the fully adjusted Model 3 [53]. To explore the interactions with hs-CRP and SII, subgroup analyses were also conducted with stratification by age (<50 years or ≥50 years), sex, race, BMI, smoking status, diabetes mellitus, CKD, hypertension, CHD, and hyperlipidemia.

All tests were two-tailed with a significance level of α = 0.05. Continuous variables are presented as weighted mean values with 95% CIs, while categorical variables are presented as frequencies and weighted percentages (%).

## 3 RESULTS

### 3.1 Participants’ Baseline Characteristics

Overall, 4,870 participants (mean age 37.97 ± 12.42 years) were included in this study; 51.11% were women and 48.89% were men. The overall prevalence of sarcopenic obesity was 6.65%, whereas the prevalence of sarcopenia alone was 9.75%.

The hs-CRP concentration was divided into quartiles as follows: <0.67 mg/L (reference), ≥0.67 to <1.63 mg/L, ≥1.63 to <3.83 mg/L, and ≥3.83 mg/L. SII was also categorized into quartiles as follows: <319.42 × 10^9^ cells/L (reference), ≥319.42 to <441.67 × 10^9^ cells/L, ≥441.67 to <609.08 × 10^9^ cells/L, and ≥609.08 × 10^9^ cells/L. The characteristics of the participants according to inflammatory biomarker quartiles are shown in Table **1**. Participants in the highest SII quartile were more likely to be female (61.16% *vs.* 41.83%) and non-Hispanic White (36.25% *vs.* 22.13%) and to have higher educational attainment (some college or AA degree and above: 34.01% *vs.* 30.34%) than those in the lowest SII quartile. Participants in the highest SII quartile were also more likely to have a lower income (13.24% *vs.* 7.30%), diabetes mellitus (12.13% *vs.* 9.91%), hypertension (28.37% *vs.* 25.10%), and CKD (2.60% *vs.* 1.22%), and they were also more likely to be smokers (23.79% *vs.* 17.57%).

Participants in the highest hs-CRP quartile were more likely to be older (>50 years: 24.22% *vs.* 14.64%), female (64.81% *vs.* 44.10%), and non-Hispanic White (31.27% *vs.* 30.21%), and were more highly educated (some college or AA degree: 34.34% *vs*. 30.68%) than those in the lowest hs-CRP quartile. Participants in the highest hs-CRP quartile were also more likely to be married or cohabiting (60.42% *vs.* 51.05%) and smokers (21.94% *vs.* 17.82%) and to have a lower income (21.32% *vs*. 19.25%), diabetes mellitus (20.45% *vs.* 4.19%), hypertension (35.42% *vs.* 14.56%), CVD (14.11% *vs.* 5.27%), and CKD (2.82% *vs.* 0.67%).

### 3.2 Association between Sarcopenia and Inflammatory Biomarkers

The associations between sarcopenia and the inflammatory biomarkers in Models 1, 2, and 3 are summarized in Table **2**. Among the participants with sarcopenia, inflammatory biomarkers demonstrated an association with sarcopenia. In the fully adjusted model (Model 3), the positive association remained (SII Model 3: OR 1.05, 95% CI 1.02–1.08, *p* = 0.0011; hs-CRP Model 3: OR 1.02, 95% CI 1.01–1.03, *p* < 0.0001). Sensitivity analysis showed that in the fully adjusted model (Model 3), participants in the highest SII quartile (OR 1.94, 95% CI 1.41–2.68, *p* < 0.0001) and the highest hs-CRP quartile (OR 6.09, 95% CI 3.89–9.53, *p* < 0.0001) had a significantly higher risk of developing sarcopenia.

### 3.3 Association between Sarcopenic Obesity and Inflammatory Biomarkers

The associations between sarcopenic obesity and the inflammatory biomarkers in Models 1, 2, and 3 are summarized in Table **3**. The inflammatory biomarkers showed an association with sarcopenic obesity. In the fully adjusted model (Model 3), the positive association remained (SII Model 3: OR 1.04, 95% CI 1.00–1.08, *p* = 0.0448; hs-CRP Model 3: OR 1.03, 95% CI 1.02–1.04, *p* < 0.0001). Sensitivity analysis showed that in the fully adjusted model (Model 3), participants in the highest SII quartile (OR 1.82, 95% CI 1.24–2.67, *p* < 0.0022) and those in the highest hs-CRP quartile (OR 24.69, 95% CI 8.99–67.78, *p* < 0.0001) had a significantly higher risk of developing sarcopenic obesity.

### 3.4 Subgroup Analysis

We conducted a subgroup analysis to explore the consistency in the relationship between the inflammatory biomarkers and sarcopenia/sarcopenic obesity across different subgroups (Figs. **2a**-**d**). The population was stratified into subgroups by age, sex, race, BMI, hypertension, diabetes mellitus, smoking status, hyperlipidemia, CVD, and CKD.

## 4 DISCUSSION

This study investigated the associations of two inflammatory biomarkers (hs-CRP and SII) with sarcopenia and sarcopenic obesity in younger individuals [53, 54]. This cross- sectional analysis of 4,870 US adults based on NHANES data revealed that high hs-CRP and high SII significantly increased the risk of developing sarcopenia and sarcopenic obesity among people aged ≥18 years. The multivariate logistic regression analysis showed that participants in the highest quartiles of these inflammatory biomarkers had a significantly higher risk of developing sarcopenia and sarcopenic obesity than those in the lowest quartiles. These findings suggest that higher hs-CRP and SII are positively associated with the incidence of sarcopenia and sarcopenic obesity in the younger population, indicating that those with systemic inflammation are more likely to develop these conditions than those without [55].

These results are consistent with those of previous studies in older populations. For example, a cross-sectional study conducted in China showed that in men aged ≥65 years, those with sarcopenic obesity had a significantly higher serum hs-CRP level than those without [56, 57]. Similarly, Kim *et al*. reported that the serum CRP level was independently associated with sarcopenic obesity in Korean women [58]. A meta-analysis showed that older individuals with high SII had a 1.58-fold higher risk of developing sarcopenia than those with low SII, suggesting that elevated SII is a risk factor for sarcopenia [59, 60]. Furthermore, a cross-sectional study showed that elevated SII in middle-aged and older adults was associated with an increased risk of sarcopenic obesity [61]. Another cross-sectional study of 4,224 Chinese adults aged 45–90 years revealed that higher SII was significantly associated with an increased prevalence of sarcopenia [62]. However, most of these studies focused only on the elderly. Consequently, data in younger populations remain scarce. Our analysis extends the conclusions of previous studies by providing insights into the relationships of these two inflammatory biomarkers with sarcopenia and sarcopenic obesity in younger adults.

Several mechanisms may explain the positive associations between inflammatory biomarkers and sarcopenia/sarcopenic obesity. Both obesity and sarcopenia are commonly characterized by chronic pro-inflammatory states, which may disrupt metabolic processes and affect the function of both adipose and muscle tissues [63-66]. Inflammation affects appetite regulation, leading to weight changes. Inflammation may also cause anorexia and fat loss in some individuals, which can be accompanied by sarcopenia [67, 68]. Additionally, inflammation is particularly relevant to sarcopenic obesity and is exacerbated by alterations in glucose metabolism, insulin resistance, oxidative stress, and inflammatory cytokine production [69-71]. Numerous studies have explored the association between inflammation and muscle metabolism; however, the mechanism underlying this association remains to be clarified. Prolonged and sustained inflammatory responses increase muscle protein breakdown and decrease protein synthesis, leading to muscle loss and reduced muscle quality [72, 73]. Individuals with higher systemic inflammation tend to have higher fat mass [74]. Adipose tissue produces tumor necrosis factor-α, monocyte chemoattractant protein-1, interleukin-6, and CRP, which affect insulin resistance and anabolic hormone secretion, thereby promoting the development of sarcopenic obesity [5, 75].

To our knowledge, this study is the first to report the associations of inflammatory biomarkers with sarcopenia and sarcopenic obesity in younger adults, offering reliable data and more options to screen for sarcopenia among high-risk community populations. The strengths of this study are in its design and statistical analysis. We evaluated two inflammatory biomarkers that can be easily measured using routine clinical tests, allowing us to identify their associations with sarcopenia and sarcopenic obesity, which could be easily translated into clinical practice.

This study also has some limitations that should be acknowledged. First, owing to the cross-sectional study design, the causal relationships between exposures and outcomes could not be explored. Second, the dataset contained limited survey variables. For instance, this iteration of the NHANES did not include muscle strength data, which is important when investigating sarcopenia.

## CONCLUSION

Given the positive associations between inflammatory biomarkers and sarcopenia/sarcopenic obesity, this study confirmed the utility of hs-CRP and SII as prospective indicators for predicting the development of sarcopenia and sarcopenic obesity in younger adults. Regular monitoring of hs-CRP and SII may help clinicians identify individuals at a higher risk of developing sarcopenia and sarcopenic obesity, facilitating the early implementation of targeted interventions. However, the specific molecular mechanisms underlying these associations remain unclear. Future research should focus on elucidating the potential biological pathways linking hs-CRP and SII to sarcopenia and sarcopenic obesity and evaluating whether interventions that reduce these biomarkers can effectively decrease the incidence of these conditions.

## Figures and Tables

**Fig. (1):**
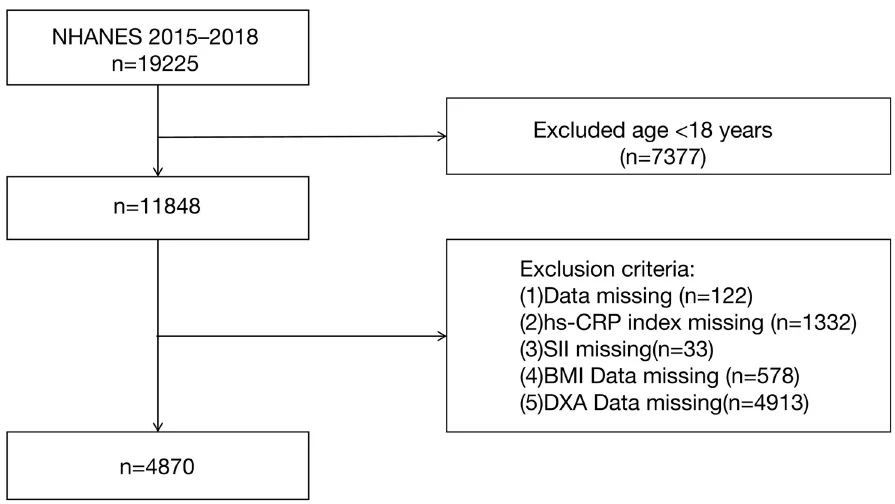
Flowchart of participant selection. **Abbreviations**: BMI, body mass index; DXA, dual-energy X-ray absorptiometry; hs-CRP, high- sensitivity C-reactive protein; NHANES, National Health and Nutrition Examination Survey; SII, systemic immune–inflammation index.

**Fig. (2):**
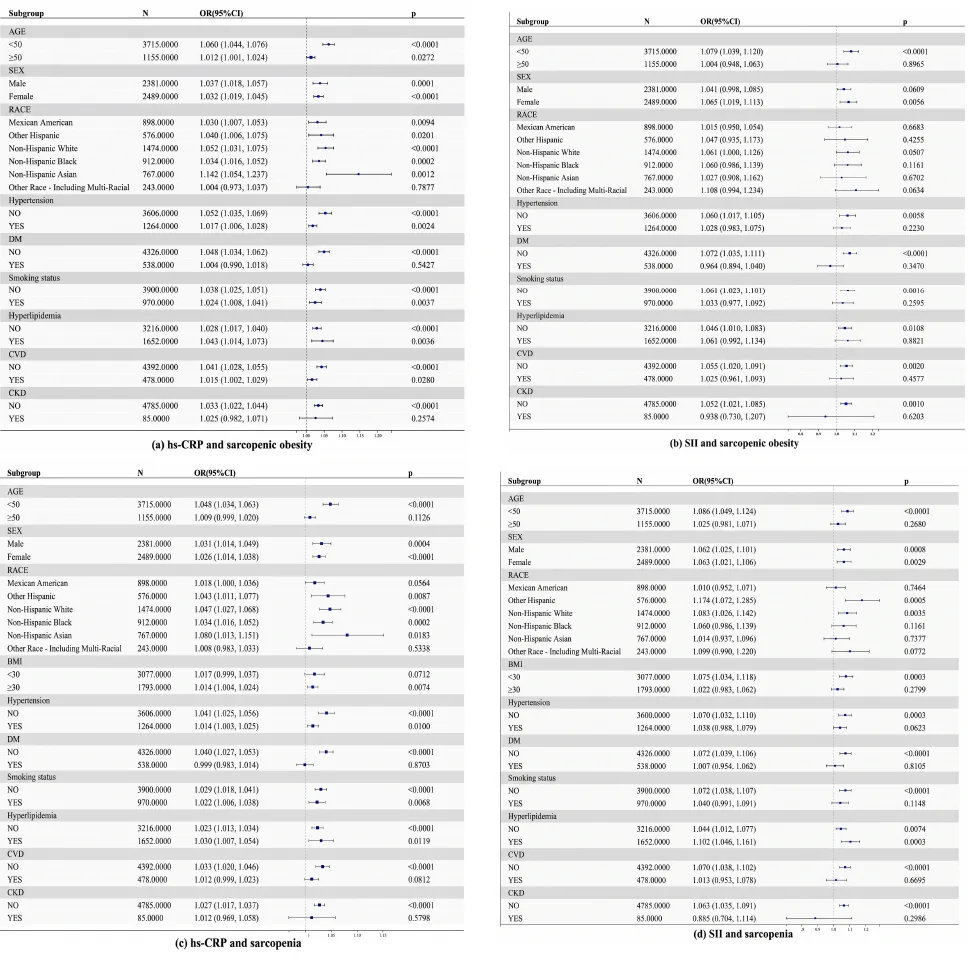
Results of the subgroup analysis. Association between (**a**) hs-CRP and sarcopenic obesity, (**b**) SII and sarcopenic obesity, (**c**) hs-CRP and sarcopenia, and (**d**) SII and sarcopenia. **Abbreviations:** BMI, body mass index; CI, confidence interval; CKD, chronic kidney disease; CVD, cardiovascular disease; DM, diabetes mellitus; hs-CRP, high-sensitivity C-reactive protein; OR, odds ratio; SII, systemic immune–inflammation index.

**Table 1:** Participants’ characteristics according to inflammatory biomarker quartiles.

Variable	Overall	SII (×10^9^ cells/L)				*p*	hs-CRP (mg/L)				*p*
n (%)	4,870 (100%)	<319.42 (Quartile 1)	≥319.42 to <441.67 (Quartile 2)	≥441.67 to <609.08 (Quartile 3)	≥609.08 (Quartile 4)	-	<0.67 (Quartile 1)	≥0.67 to <1.63 (Quartile 2)	≥1.63 to <3.83 (Quartile 3)	≥3.83 (Quartile 4)	-
		1,315 (27%)	1,193 (24.5%)	1,206 (24.75%)	1,156 (23.74%)	-	1,195 (24.54%)	1,222 (25.09%)	1,177 (24.17%)	1,276 (26.2%)	-
Age (years)	-	-	-	-	-	<0.001	-	-	-	-	<0.001
18–40	2,713 (55.71%)	727 (55.29%)	659 (55.24%)	688 (57.05%)	639 (55.28%)	-	814 (68.12%)	682 (55.81%)	589 (50.04%)	628 (49.22%)	-
41–50	1,110 (22.79%)	257 (19.54%)	264 (22.13%)	285 (23.63%)	304 (26.30%)	-	206 (17.24%)	260 (21.28%)	305 (25.91%)	339 (26.57%)	-
>50	1,047 (21.50%)	331 (25.17%)	270 (22.63%)	233 (19.32%)	213 (18.43%)	-	175 (14.64%)	280 (22.91%)	283 (24.04%)	309 (24.22%)	-
Sex	-	-	-	-	-	<0.001	-	-	-	-	<0.001
Female	2,489 (51.11%)	550 (41.83%)	575 (48.20%)	657 (54.48%)	707 (61.16%)	-	527 (44.10%)	565 (46.24%)	570 (48.43%)	827 (64.81%)	-
Male	2,381 (48.89%)	765 (58.17%)	618 (51.80%)	549 (45.52%)	449 (38.84%)	-	668 (55.90%)	657 (53.76%)	607 (51.57%)	449 (35.19%)	-
Race	-	-	-	-	-	<0.001	-	-	-	-	<0.001
Mexican American	898 (18.44%)	207 (15.74%)	245 (20.54%)	219 (18.16%)	227 (19.64%)	-	146 (12.22%)	236 (19.31%)	259 (22.01%)	257 (20.14%)	-
Other hispanic	576 (11.83%)	128 (9.73%)	129 (10.81%)	173 (14.34%)	146 (12.63%)	-	116 (9.71%)	148 (12.11%)	135 (11.47%)	177 (13.87%)	-
Non-hispanic white	1,474 (30.27%)	291 (22.13%)	370 (31.01%)	394 (32.67%)	419 (36.25%)	-	361 (30.21%)	353 (28.89%)	361 (30.67%)	399 (31.27%)	-
Non-hispanic black	912 (18.73%)	397 (30.19%)	205 (17.18%)	159 (13.18%)	151 (13.06%)	-	229 (19.16%)	195 (15.96%)	224 (19.03%)	264 (20.69%)	-
Non-hispanic Asian	767 (15.75%)	234 (17.79%)	189 (15.84%)	195 (16.17%)	149 (12.89%)	-	286 (23.93%)	236 (19.31%)	137 (11.64%)	108 (8.46%)	-
Other race (including multi-racial)	243 (4.99%)	58 (4.41%)	55 (4.61%)	66 (5.47%)	64 (5.54%)	-	57 (4.77%)	54 (4.42%)	61 (5.18%)	71 (5.56%)	-
Educational attainment	-	-	-	-	-	0.002	-	-	-	-	<0.001
<9^th^ Grade	329 (7.32%)	84 (6.98%)	106 (9.57%)	77 (6.99%)	62 (5.73%)	-	60 (5.83%)	87 (7.66%)	88 (7.94%)	94 (7.70%)	-
9-11^th^ Grade (including 12^th^ Grade with no diploma)	483 (10.75%)	115 (9.56%)	113 (10.20%)	113 (10.25%)	142 (13.12%)	-	88 (8.54%)	110 (9.68%)	125 (11.27%)	160 (13.11%)	-
High school graduate/GED or equivalent	1,016 (22.60%)	276 (22.94%)	236 (21.30%)	256 (23.23%)	248 (22.92%)	-	211 (20.49%)	255 (22.45%)	264 (23.81%)	286 (23.44%)	-
Some college or AA degree	1,460 (32.48%)	365 (30.34%)	365 (32.94%)	362 (32.85%)	368 (34.01%)	-	316 (30.68%)	350 (30.81%)	375 (33.81%)	419 (34.34%)	-
College graduate or above	1,207 (26.85%)	363 (30.17%)	288 (25.99%)	294 (26.68%)	262 (24.21%)	-	355 (34.47%)	334 (29.40%)	257 (23.17%)	261 (21.39%)	-
Marital status	-	-	-	-	-	0.416	-	-	-	-	<0.001
Not married	2,091 (42.94%)	547 (41.60%)	501 (41.99%)	531 (44.03%)	512 (44.29%)	-	585 (48.95%)	507 (41.49%)	494 (41.97%)	505 (39.58%)	-
Married or living with partner	2,779 (57.06%)	768 (58.40%)	692 (58.01%)	675 (55.97%)	644 (55.71%)	-	610 (51.05%)	715 (58.51%)	683 (58.03%)	771 (60.42%)	-
PIR	-	-	-	-	-	0.156	-	-	-	-	0.005
High income	1,094 (22.46%)	314 (28.70%)	262 (23.95%)	277 (25.32%)	241 (22.03%)	-	302 (80.75%)	312 (84.04%)	239 (79.86%)	241 (78.68%)	-
Medium income	2,390 (49.07%)	637 (26.65%)	584 (24.44%)	595 (24.90%)	574 (24.02%)	-	539 (22.55%)	608 (25.44%)	590 (24.69%)	653 (27.32%)	-
Low income	934 (19.18%)	232 (17.64%)	238 (19.95%)	222 (18.41%)	242 (20.93%)	-	230 (19.25%)	195 (15.96%)	237 (20.14%)	272 (21.32%)	-
Sarcopenia	-	-	-	-	-	<0.001	-	-	-	-	<0.001
No	4,395 (90.25%)	1,219 (92.70%)	1,087 (91.11%)	1,086 (90.05%)	1,003 (86.76%)	-	1,164 (97.41%)	1,126 (92.14%)	1,046 (88.87%)	1,059 (82.99%)	-
Yes	475 (9.75%)	96 (7.30%)	106 (8.89%)	120 (9.95%)	153 (13.24%)	-	31 (2.59%)	96 (7.86%)	131 (11.13%)	217 (17.01%)	-
Smoking status	-	-	-	-	-	<0.001	-	-	-	-	0.016
No	3,900 (80.08%)	1,084 (82.43%)	974 (81.64%)	961 (79.68%)	881 (76.21%)	-	982 (82.18%)	998 (81.67%)	924 (78.50%)	996 (78.06%)	-
Yes	970 (19.92%)	231 (17.57%)	219 (18.36%)	245 (20.32%)	275 (23.79%)	-	213 (17.82%)	224 (18.33%)	253 (21.50%)	280 (21.94%)	-
Diabetes mellitus	-	-	-	-	-	0.362	-	-	-	-	<0.001
No	4,326 (88.94%)	1,182 (90.09%)	1,058 (88.68%)	1,072 (88.96%)	1,014 (87.87%)	-	1,142 (95.81%)	1,135 (93.03%)	1,034 (87.93%)	1,015 (79.55%)	-
Yes	538 (11.06%)	130 (9.91%)	135 (11.32%)	133 (11.04%)	140 (12.13%)	-	50 (4.19%)	85 (6.97%)	142 (12.07%)	261 (20.45%)	-
Hypertension	-	-	-	-	-	0.188	-	-	-	-	<0.001
No	3,606 (74.05%)	985 (74.90%)	896 (75.10%)	897 (74.38%)	828 (71.63%)	-	1,021 (85.44%)	939 (76.84%)	822 (69.84%)	824 (64.58%)	-
Yes	1,264 (25.95%)	330 (25.10%)	297 (24.90%)	309 (25.62%)	328 (28.37%)	-	174 (14.56%)	283 (23.16%)	355 (30.16%)	452 (35.42%)	-
Hyperlipidemia	-	-	-	-	-	<0.001	-	-	-	-	<0.001
No	3,216 (66.06%)	801 (60.96%)	802 (67.23%)	836 (69.32%)	777 (67.27%)	-	579 (48.53%)	790 (64.65%)	872 (74.09%)	975 (76.41%)	-
Yes	1,652 (33.94%)	513 (39.04%)	391 (32.77%)	370 (30.68%)	378 (32.73%)	-	614 (51.47%)	432 (35.35%)	305 (25.91%)	301 (23.59%)	-
CVD	-	-	-	-	-	0.782	-	-	-	-	<0.001
No	4,392 (90.18%)	1,178 (89.58%)	1,075 (90.11%)	1,095 (90.80%)	1,044 (90.31%)	-	1,132 (94.73%)	1,112 (91.00%)	1,052 (89.38%)	1,096 (85.89%)	-
Yes	478 (9.82%)	137 (10.42%)	118 (9.89%)	111 (9.20%)	112 (9.69%)	-	63 (5.27%)	110 (9.00%)	125 (10.62%)	180 (14.11%)	-
CKD	-	-	-	-	-	0.051	-	-	-	-	<0.001
No	4,785 (98.25%)	1,299 (98.78%)	1,176 (98.58%)	1,184 (98.18%)	1,126 (97.40%)	-	1,187 (99.33%)	1,201 (98.28%)	1,157 (98.30%)	1,240 (97.18%)	-
Yes	85 (1.75%)	16 (1.22%)	17 (1.42%)	22 (1.82%)	30 (2.60%)	-	8 (0.67%)	21 (1.72%)	20 (1.70%)	36 (2.82%)	-
Age (years)	37.97 ± 12.42	38.19 ± 12.91	38.05 ± 12.61	37.53 ± 12.09	38.08 ± 11.99	0.558	34.13 ± 12.44	38.34 ± 12.24	39.29 ± 12.21	39.98 ± 11.99	<0.001
BMI (kg/m^2^)	28.79 ± 6.81	27.50 ± 5.96	28.51 ± 6.46	29.35 ± 7.01	29.95 ± 7.56	<0.001	24.06 ± 4.13	27.14 ± 4.83	30.25 ± 6.16	33.44 ± 7.53	<0.001

**Note:** Data are expressed as n (%) or mean ± standard deviation. Low income was defined as PIR < 1; medium income as PIR ≥ 1 to ≤ 4; and high income as PIR > 4. **Abbreviations:** BMI, body mass index; CKD, chronic kidney disease; CVD, cardiovascular disease; GED, General Educational Development; hs-CRP, high-sensitivity C-reactive protein; PIR, poverty-to-income ratio; SII, systemic immune–inflammation index.

**Table 2:** Association between inflammatory biomarkers and sarcopenia.

**Sarcopenia**	**OR (95% CI) *p* value**		
	**Model 1**	**Model 2**	**Model 3**
SII	1.06 (1.03–1.09) **<0.0001**	1.06 (1.03–1.09) **<0.0001**	1.05 (1.02–1.08) **0.0011**
**Stratified by SII quartile**			
Quartile 1	1	1	1
Quartile 2	1.24 (0.93–1.65) 0.1458	1.10 (0.82–1.49) 0.5208	1.16 (0.83–1.63) 0.3718
Quartile 3	1.40 (1.06–1.86) **0.0180**	1.33 (0.99–1.79) 0.0555	1.52 (1.09–2.10) 0.0124
Quartile 4	1.94 (1.48–2.53) **<0.0001**	1.89 (1.42–2.51) **<0.0001**	1.94 (1.41–2.68) **<0.0001**
	-	-	-
hs-CRP	1.03 (1.02–1.04) **<0.0001**	1.03 (1.02–1.04) **<0.0001**	1.02 (1.01–1.03) **<0.0001**
**Stratified by hs-CRP quartile**			
Quartile 1	1	1	1
Quartile 2	3.20 (2.12–4.84) **<0.0001**	2.56 (1.68–3.90) **<0.0001**	2.28 (1.43–3.62) **0.0005**
Quartile 3	4.70 (3.15–7.02) **<0.0001**	3.89 (2.58–5.87) **<0.0001**	3.45 (2.18–5.44) **<0.0001**
Quartile 4	7.69 (5.23–11.31) **<0.0001**	7.15 (4.79–10.67) **<0.0001**	6.09 (3.89–9.53) **<0.0001**

**Note:** Bold values indicate statistical significance. The hs-CRP quartiles were <0.67 mg/L (reference; quartile 1), ≥0.67 to <1.63 mg/L (quartile 2), ≥1.63 to <3.83 mg/L (quartile 3), and ≥3.83 mg/L (quartile 4). The SII quartiles were <319.42 × 10^9^ cells/L (reference; quartile 1), ≥319.42 to <441.67 × 10^9^ cells/L (quartile 2), ≥441.67 to <609.08 × 10^9^ cells/L (quartile 3), and ≥609.08 × 10^9^ cells/L (quartile 4). Model 1 was unadjusted; Model 2 was adjusted for age, sex, and race; and Model 3 was adjusted for age, sex, race, educational attainment, marital status, PIR, smoking status, diabetes mellitus, hypertension, CKD, CVD, and hyperlipidemia. **Abbreviations:** CI, confidence interval; CKD, chronic kidney disease; CVD, cardiovascular disease; hs-CRP, high-sensitivity C-reactive protein; OR, odds ratio; PIR, poverty-to-income ratio; SII, systemic immune–inflammation index.

**Table 3:** Association between inflammatory biomarkers and sarcopenic obesity.

**Sarcopenic Obesity**	**OR (95% CI) *p* value**		
	**Model 1**	**Model 2**	**Model 3**
SII	1.05 (1.02–1.08) **0.0012**	1.05 (1.01–1.08) **0.0060**	1.04 (1.00–1.08) **0.0448**
**Stratified by SII quartile**			
Quartile 1	1	1	1
Quartile 2	1.34 (0.94–1.89) 0.1013	1.17 (0.82–1.68) 0.3788	1.21 (0.81–1.80) 0.3505
Quartile 3	1.53 (1.09–2.15) **0.0131**	1.41 (1.00–2.00) 0.0530	1.49 (1.01–2.21) **0.0444**
Quartile 4	1.98 (1.43–2.74) **<0.0001**	1.79 (1.28–2.52) **0.0007**	1.82 (1.24–2.67) **0.0022**
hs-CRP index	1.03 (1.02–1.04) **<0.0001**	1.03 (1.02–1.04) **<0.0001**	1.03 (1.02–1.04) **<0.0001**
**Stratified by hs-CRP quartile**			
Quartile 1	1	1	1
Quartile 2	8.10 (3.46–18.99) **<0.0001**	6.34 (2.69–14.93) **<0.0001**	6.11 (2.15–17.32) **0.0007**
Quartile 3	16.61 (7.24–38.07) **<0.0001**	12.68 (5.50–29.24) **<0.0001**	12.89 (4.66–35.67) **<0.0001**
Quartile 4	32.34 (14.28–73.20) **<0.0001**	26.88 (11.78–61.36) **<0.0001**	24.69 (8.99–67.78) **<0.0001**

**Note:** Bold values indicate statistical significance. The hs-CRP quartiles were <0.67 mg/L (reference; quartile 1), ≥0.67 to <1.63 mg/L (quartile 2), ≥1.63 to <3.83 mg/L (quartile 3), and ≥3.83 mg/L (quartile 4). The SII quartiles were <319.42 × 10^9^ cells/L (reference; quartile 1), ≥319.42 to <441.67 × 10^9^ cells/L (quartile 2), ≥441.67 to <609.08 × 10^9^ cells/L (quartile 3), and ≥609.08 × 10^9^ cells/L (quartile 4). Model 1 was unadjusted; Model 2 was adjusted for age, sex, and race; and Model 3 was adjusted for age, sex, race, educational attainment, marital status, PIR, smoking status, diabetes mellitus, hypertension, CKD, CVD, and hyperlipidemia. **Abbreviations:** CI, confidence interval; CKD, chronic kidney disease; CVD, cardiovascular disease; hs-CRP, high-sensitivity C-reactive protein; OR, odds ratio; PIR, poverty-to-income ratio; SII, systemic immune–inflammation index.

## Data Availability

All data generated or analyzed during this study are included in this published article.

## LIST OF ABBREVIATIONS

ASM: Appendicular Skeletal Muscle Mass
BMI: Body Mass Index
CHD: Coronary Heart Disease
CI: Confidence Interval
CKD: Chronic Kidney Disease
CRP: C-reactive Protein
CVD: Cardiovascular Disease
DXA: Dual-energy X-ray Absorptiometry
GED: General Educational Development
hs-CRP: High-sensitivity C-reactive Protein
NHANES: National Health and Nutrition Examination Survey
OR: Odds Ratio
PIR: Poverty-to-income Ratio
SII: Systemic Immune–inflammation Index

## STANDARDS OF REPORTING

STROBE guidelines were followed.
